# Total and Segmental Colon Transit Time Study in Functional Constipation: Comparison With Healthy Subjects

**DOI:** 10.14740/gr642w

**Published:** 2015-02-14

**Authors:** Prasad A. Bhate, Jatin A. Patel, Pathik Parikh, Meghraj A. Ingle, Anniruddha Phadke, Prabha D. Sawant

**Affiliations:** a Department of Gastroenterology, Lokmanya Tilak Municipal Medical College and Hospital, Sion, Mumbai, India

**Keywords:** Functional constipation, Segmental colon transit time, Total colon transit time

## Abstract

**Background:**

Constipation is a common problem worldwide. Constipation can be primary or secondary. Primary constipation is subdivided in slow transit constipation, normal transit constipation, and dyssynergic defecation. Colon transit time (CTT) is the most basic and primary tool in evaluating disorders of colonic motility. CTT helps to differentiate between types of constipation and plan the treatment.

**Methods:**

Fifty functional constipation patients and 25 healthy controls were asked to ingest four gelatin capsules (each containing five radio-opaque markers) at 0, 12 and 24 hours. An abdominal X-ray was taken at 36 hours. Total or segmental CTT was measured after calculating the number of markers remaining in each segment at 36 hours on abdominal X-ray.

**Results:**

Mean CTT in healthy controls in our study was 15.4 hours which is shorter than western population. Total CTT is significantly higher in constipation group (23 hours) compared to healthy subjects (15.4 hours). Transit time in right segment was significantly high in constipation group than healthy population (14.2 vs. 8.3 hours). Total as well as segmental transit times are slightly higher in females as compared to males in both the groups, however not statistically significant. To the best of our knowledge, there are no studies from India that compared the CTTs in functional constipation and healthy controls.

**Conclusion:**

Radio-opaque marker study for CTT is a simple and reliable technique for evaluation of constipation. Patients with functional constipation have significantly longer CTT than healthy population. Total CTT is much less in this study population compared to west. There is need to establish standards for slow colon transit.

## Introduction

Constipation is a common problem worldwide [1]. Constipation can be primary or secondary. Primary constipation is subdivided into slow transit, normal transit constipation and dyssynergic defecation. Though in most patients constipation is caused by irritable bowel syndrome (IBS) in the West, in about half of these patients, it is caused either by slow transit constipation (STC) or fecal evacuation disorders (FEDs) [1]. There are various methods for investigating constipation like plain abdominal radiograph, contrast enema, defecating proctogram, measurement of colonic transit time and anorectal manometry [1].

Out of the above tests, measurement of colon transit time (CTT) is the most basic and primary tool in evaluating disorders of colonic motility. CTT helps to differentiate between types of constipation and plan the treatment. Various methods are available for measuring CTT. The standard technique is radio-opaque marker test which is a most widely used method because it is easy to perform and cost effective. Only disadvantage of this method is radiation exposure. Radionuclide scintigraphy and wireless motility capsules are other techniques used to measure CTT [2].

In a country like India, it is not easy to investigate the patient of constipation because of lack of widespread availability of anorectal manometry and barium defecography. So CTT becomes an important test for the management of patients with constipation.

CTT test protocol in western countries is use of 20 radio-opaque markers to be given at 0, 24 and 48 h each, followed by an abdominal X-ray at 72 h [3-5]. However, Indians have rapid gut transit, so above protocol may not be suitable for measuring CTT. To overcome this problem, some investigators have attempted to decrease the interval between ingestion of markers and abdominal X-ray. Nabar et al developed a protocol in which 20 radio-opaque markers were given at 0, 9 and 18 h followed by abdominal X-ray at 27 h [6]. Other study from India used a protocol of administering 20 radio-opaque markers at 0, 12 and 24 h each followed by an abdominal X-ray at 12, 24 and 36 h [7]. Recently Ghoshal et al reported a different protocol in which radio-opaque markers were given at 0, 12 and 24 h followed by X-ray taken at 36 and 60 h [8].

There is paucity of CTT studies from India in patients of functional constipation.

## Methods

### Subjects

Patients, diagnosed as functional constipation (Rome 3 criteria), attending GI OPD of a large tertiary public hospital were included in study. Diagnostic work-up, including colonoscopy, barium enema and/or anorectal manometry as well as blood tests to rule out hypothyroidism, hypercalcemia was performed. Patients with abnormal findings suggestive of an organic disease in any of the investigations were excluded. Other exclusion criteria were pregnancy, severe medical illness, alcoholism, patients taking drugs affecting motility and history of major abdominal surgery. Controls were healthy subjects without any gastrointestinal complaints.

### Measurement of CTT

CTT was measured by using radio-opaque markers. Patients were asked to ingest four gelatin capsules (each containing five radio-opaque markers) at 0, 12 and 24 h. Abdominal X-ray was taken at 36 h from the time of ingestion of first dose of markers. Localization of markers on X-ray was done by identifying body structures as described in a study by Arhan et al [9]. Markers located on right of the vertebral spinous processes above a line from the fifth vertebra to the right pelvic outlet were assigned to the right colon. Markers to the left of the vertebral spinous processes and above an imaginary line from the fifth lumbar vertebra to the anterior superior iliac crest were assigned to the left colon. And markers inferior to a line from the pelvic brim on the right and the superior iliac crest on the left were judged to be in the right sigmoid colon and rectum [10].

Total or segmental CTT was measured after calculating the number of markers remaining in each segment at 36 h on abdominal X-ray [11].

CTT (or segmental transit time) = 12/20 × (n) hours, where n is the sum of the markers on the X-ray film (or in the delineated segments).

Subjects were asked to follow their usual daily routine and diet, to avoid unusual intensive physical activity and alcohol intake.

### Statistical analysis

Unpaired Student’s *t*-test was used to compare the mean transit time between the two groups. All patients completed the informed consent form. The study protocol was approved by the ethics committee of our institute.

## Results

Fifty functional constipation patients and age and sex matched 25 healthy controls were enrolled (Tables 1-3). The duration of constipation ranged between 8 months to 20 years in FC patients.

**Table 1:** Basic Characteristics

	Functional constipation	Healthy subjects
Age (SD)	33.3 years (11.8)	32.5 years (10.3)
Gender (M:F)	26:24	12:13
BMI (SD)	22 kg/m^2^ (1.3)	21.2 kg/m^2^ (1.7)

**Table 2:** Mean Transit Time in Hours

	Functional constipation	Healthy subjects	
Total colon transit time (h)	23	15.4	P < 0.05
Segmental transit time (h)			
Right	14.2	8.3	P < 0.05
Left	6.5	4	n.s.
Rectosigmoid	5	3.5	n.s.

**Table 3:** Mean Transit Time in Male and Female

	Functional constipation		Healthy subjects		
	Male	Female	Male	Female	
Total colon transit time (h)	21	25	14.2	17	n.s.
Segmental transit time (h)					
Right	12.9	15	7.8	8.5	n.s.
Left	5.8	6.8	3.7	4.6	n.s.
Rectosigmoid	4.7	5.9	3	4	n.s.

## Discussion

Radio-opaque marker test is a simple and convenient method to measure CTT. There is a paucity of literature regarding CTT study in functional constipation patients in India. In the present study, multiple capsule and single film technique was used to ensure patient compliance and minimize radiation exposure [10]. As gut transit is faster in India, it is recommended to shorten the duration between marker ingestion and X-ray abdomen [11].

Mean CTT in healthy controls in our study was 15.4 h which is shorter than western population [12, 13]. Our findings are consistent with other studies from India [6, 8, 12]. This is probably attributed to higher fiber content in Indian diet. The average stool frequency in India is one per day [14]. In western literature, the transit times in right, left and rectosigmoid colon are similar [10]. In our study, the transit time in right segment is higher as compared to left and rectosigmoid region.

We compared the CTTs in patients with functional constipation and healthy controls. We found that total CTT is significantly higher in constipation group (23 h) compared to healthy subjects (15.4 h). Transit time in right segment was significantly high in constipation group than healthy population (14.2 vs. 8.3 h). The difference of left segment and rectosigmoid transit time between both groups was not significant.

Gender comparison of transit times shows that total as well as segmental transit times are slightly higher in females as compared to males in both the groups, however not statistically significant. One study suggested that left segmental transit time was higher in females [15] and suggested progesterone may be responsible for prolongation of transit time in females.

To the best of our knowledge, there are no studies from India that compared the CTTs in functional constipation and healthy controls. So our study provides an important finding that patients with functional constipation have longer total and right segmental transit time in comparison to healthy controls. Limitation of our study is that normal range of transit time in healthy population in India is not established. Also the colonic transit is affected by age as well as gender. Rarely multiple markers may stick to one another and produce difficulty in reading the film.

In conclusion, radio-opaque marker study for CTT is a simple and reliable technique for evaluation of constipation. Patients with functional constipation have significantly longer CTT than healthy population. Total CTT is much less in this study population compared to west. There is need to establish standards for slow colon transit.
